# Double-Network Hydrogels Reinforced with Covalently Bonded Silica Nanoparticles via 1-Ethyl-3-(3-dimethylaminopropyl)carbodiimide Chemistry

**DOI:** 10.1021/acsomega.2c05169

**Published:** 2022-11-18

**Authors:** Ali A. Mohammed, Nicholas Groth Merrild, Siwei Li, Alessandra Pinna, Julian R. Jones

**Affiliations:** † Dyson School of Design Engineering, Imperial College London, SW7 9EG London, U.K.; ‡ Department of Materials, Imperial College London, SW7 2AZ London, U.K.; § Visiting Specialist Services Academy Ltd, Office 6.072 6th Floor, First Central 200, 2 Lakeside Drive, London NW10 7FQ, U.K.; ∥ The Francis Crick Institute, London NW11AT, U.K.

## Abstract

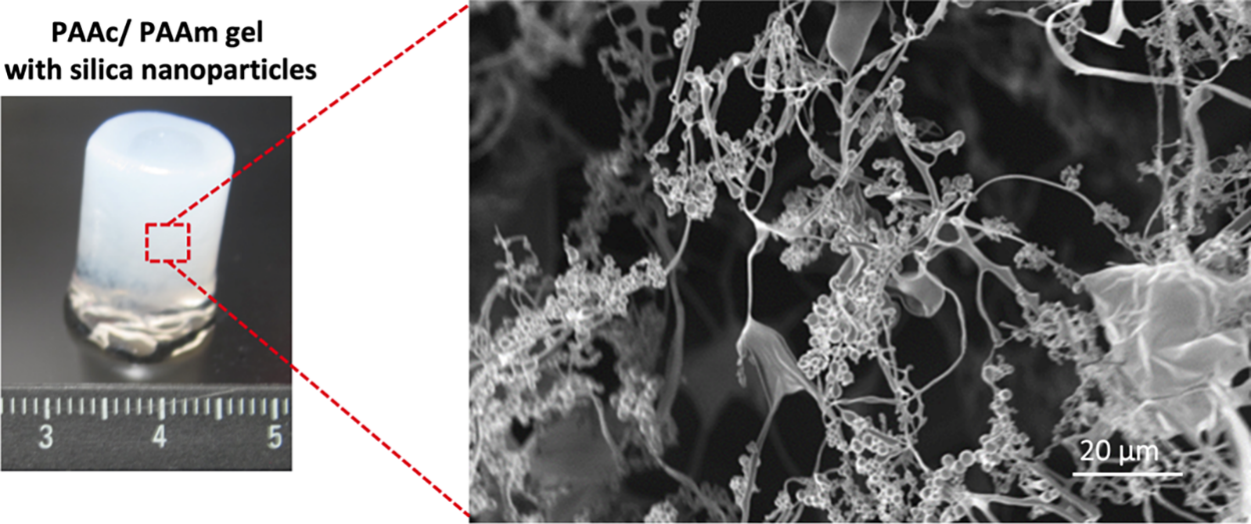

Hydrogels have progressed from single-network materials with low mechanical integrity to double-network hydrogels (DNHGs) with tough, tunable properties. In this work, we introduce a nanocomposite structure into the first network of a DNHG. Amine-functionalized silica nanoparticles (ASNPs) were covalently cross-linked by forming amide bonds through the carboxylic groups of polyacrylic acid (PAAc) in the first network. DNHGs with varying sizes of ASNPs (50, 100, and 150 nm) and varying concentrations (2.5, 10, 20, and 40 wt %) were explored and compared to a control without a nanocomposite structure. Compressive strengths improved from 0.10 MPa for the control to a maximum of 1.28 MPa for the PAAc/PAAm DNHGs. All hydrogels experienced increased resistance to strain with a maximum of 74% compared to 45% for the control. SEM images of freeze-dried gels showed that ASNPs were integrated into the gel mesh. Nanoparticle retention was calculated using thermal gravimetric analysis (TGA) with improved retention values for larger ASNPs. New DNHG composites have been formed with improved mechanical properties and a potential use in tissue engineering and biomaterial applications.

## Introduction

Hydrogels are soft and wet materials that are made up of cross-linked polymeric macromolecules and water. They have the ability to hold up to more than 90% water while maintaining their structural integrity. The characteristic similarities between hydrogels and soft tissues have drawn attention to this class of material as biomaterials. The viscoelastic polymer networks of hydrogels resemble the network structure of the extracellular matrix (ECM) found in biological tissue that is composed of more than 60% water. Therefore, a lot of focus has shifted toward developing hydrogel-based biomaterials to mimic native human tissue and facilitate tissue repair, replacement, and regeneration. For example, load-bearing tissue such as hyaline cartilage, which is made of approximately 75% water, exhibits a compressive fracture stress of 36 MPa^1,2^ that allows cartilage to sustain daily cyclical compressions. The mechanical properties of hydrogels remain to be a challenge for these applications. Conventional hydrogels, which are composed of single hydrophilic polymer networks, are usually soft, brittle, and weak, limiting their progression and success in clinical applications.^3^ They often have mechanical and tensile properties that are sub-megapascal with strains of less than 100%. Due to their highly hydrated states and low polymer density relative to their water content, hydrogels exhibit low mechanical strength and weak structural integrity. Another reason for their low mechanical properties is the heterogeneity of the polymer network structure that is formed during gelation.^3−5^ Hydrogels with heterogeneous polymer networks fail at low forces as the force applied concentrates around the shortest chains. Therefore, several new types of hydrogels have been developed to improve the mechanical performance of hydrogels by dispersing the applied load within the microstructure to reduce crack propagation. These new hydrogels include double-network hydrogels (DNHGs),^3,6−8^ photoactive hydrogels,^9−11^ nanocomposite hydrogels,^12−16^ slide-ring gels,^17−19^ and supramolecular polymer network gels^20^ among several other unique hydrogels.^21−24^

Of those examples, DNHGs have made the largest strides in terms of improving mechanical properties. DNHGs consist of two separate and contrasting polymeric networks that result in a synergistic effect of the two polymers.^6^ They have shown the ability to hold high water content while maintaining mechanical strength and toughness. The first network normally consists of a tightly cross-linked rigid polyelectrolyte polymer, while the second network is made of a sparsely cross-linked neutral polymer, typically acrylamide.^3,6,25^ Gong et al.’s original DNHG achieved exceptional mechanical properties with a fracture stress of 20 MPa and Young’s Modulus of 0.3 MPa with water content over 90%.^6^

DNHGs have progressed more with the introduction of nanocomposite structures that provide unique microstructures that further enhance the mechanical properties of the hydrogels. Nanocomposite hydrogels consist of nanoparticles that are integrated into the polymer networks during hydrogel synthesis in water. Several types of nanoparticles have been used in nanocomposite gels, such as silica nanoparticles (SNPs),^7,13^ copper nanopowder,^15,26^ laponite clay,^15^ nanoceria,^12,27^ and nanocellulose crystals.^14,28,29^ Nanoparticles enable unique properties in the hydrogels, including internal physical reinforcement to external strain, enhanced topography for cellular attachment, conductivity, bacterial resistance, antioxidation, magnetic responsiveness, and a potential for electrical signals and sensing.^3,7,30−32^ The addition of nanoparticles with functional chemical groups on their surface can result in covalent or ionic cross-links with the polymer network of the hydrogel or adsorption of the polymer chains by being entrapped within the hydrogel network.

These improvements in the physical and biochemical as well as the mechanical properties of hydrogels through the addition of nanocomposite structures have found use in a wide range of biomedical and tissue engineering applications.^33^ These include adhesives for wound healing,^30^ implantable bio-receptive scaffolds,^32^ flexible conductive sensors, and bio-wearables.^33^ For example, in tissue engineering, the application of hydrogels is often limited due to different factors including poor mechanical and limited cell attachment sites,^34^ and optical properties.^35^ However, the addition of nanocomposites addresses these limitations by enhancing the mechanical properties,^3^ which provides cellular adhesion sites for improved bio-receptivity,^34,36^ delivery of growth factors to support cell growth,^36^ increased thermal stability,^37^ and self-healing properties and promotes stem cell differentiation.^32^ Further, conventional hydrogels used for drug-delivery systems often release drugs in an uncontrolled and unpredictable manner.^38^ Nanocomposite structures provide control over the cross-linking density, microporosity, stimulus responsiveness, and mechanical properties of the hydrogel, providing more accurate long-term control for drug release.^38^

However, nanocomposite structures can also cause detrimental effects on hydrogels if they are not incorporated carefully: for an inhomogeneous distribution, particle agglomeration can lead to stress concentrations and the lack of covalent links between polymer strands and nanoparticles can cause mechanical properties to be lower than the theoretical combination of its parts.^16,32,39−41^ To help evade these problems, it is important that nanoparticle and polymer interactions are robust. This can be achieved by functionalizing the surface of the nanoparticles with chemical groups that are able to directly create covalent links with the polymers in the hydrogel. In our previous studies, amine-functionalized nanoceria (ANC) were used as initiators for a redox surface-graft polymerization. A PAMPS first network was grafted on the surface of ANCs followed by a PAAm second network, resulting in enhanced mechanical properties of an ANC double-network hydrogel.^12^ However, this method requires a thermal process and constant sonication to initiate the polymerization. Functional silica nanoparticles can instead be used at room temperature or through photopolymerization when a suitable initiating system is chosen, providing more versatility to the synthesis technique of the gels.

In this work, acrylic acid was chosen as the polyelectrolyte for the first network and acrylamide was the neutral polymer for the second network. Polyacrylic acid is a versatile polymer that is commonly used in both tissue engineering and industrial engineering. PAAc has in its structure terminal carboxylic groups that can form covalent amide cross-links through carbodiimide chemistry through −NH_2_ groups. Amine-functionalized silica nanoparticles (ASNPs) with varying diameters (50, 100, and 150 nm) and loading concentrations (2.5, 10, 20, and 40 wt %) will be used to cross-link the PAAc first network. The objective was to use carbodiimide chemistry to form amide bonds between the carboxylic group of PAAc and the amine groups of the ASNPs. The hypothesis was that the first-network gel composed of ASNP-PAAc will subsequently be swollen and polymerized in a second network monomer solution to form a nanocomposite double-network hydrogel.

## Results and Discussion

### Amine-Functionalized Silica Nanoparticles (ASNPs)

**Figure 1:**
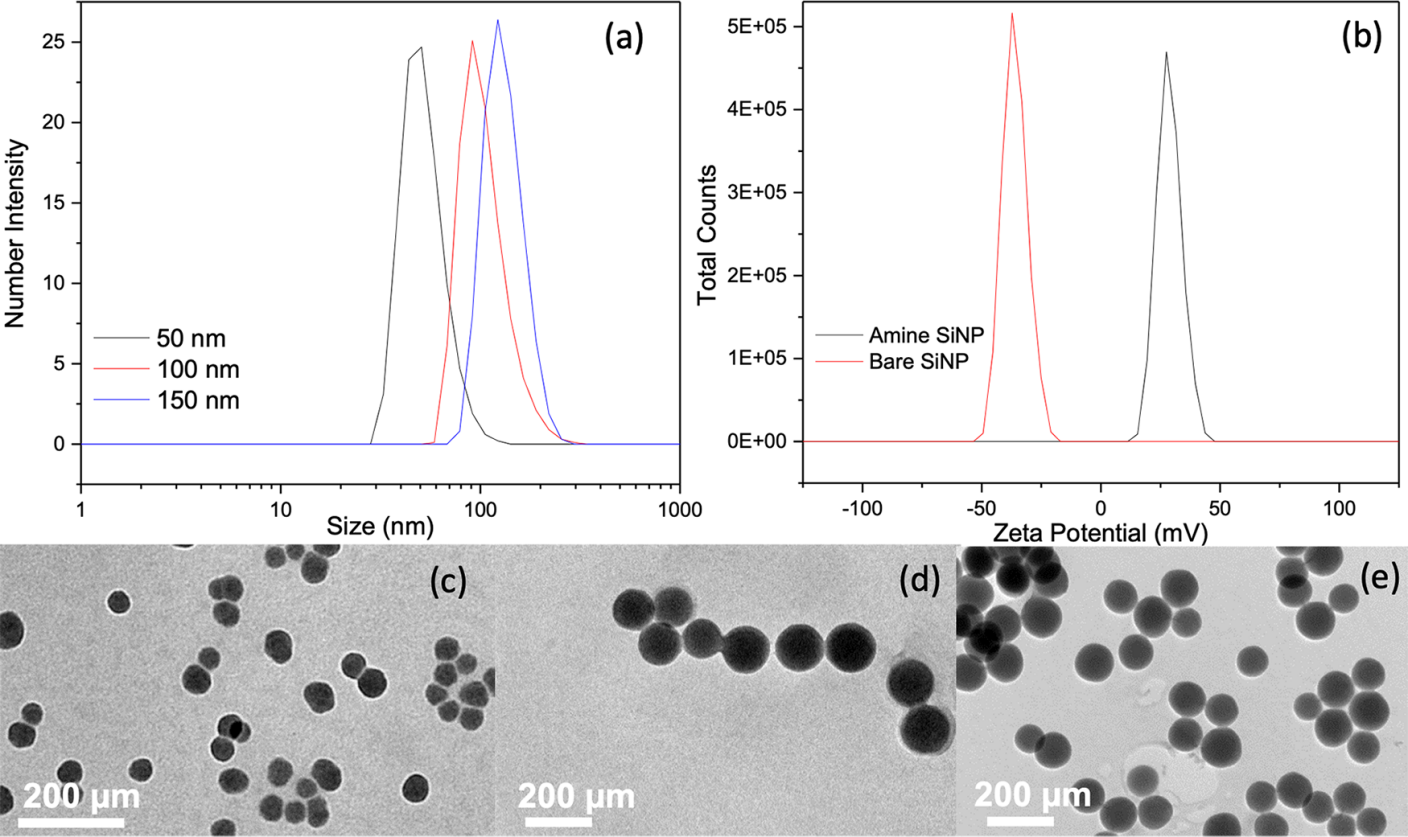
(a) DLS graph showing size profile for 50, 100, and 150 nm amine-functionalized silica nanoparticles (ASNPs) and (b) zeta potential graph showing bare and amine-functionalized silica nanoparticle peaks. TEM images of ASNPs with mean modal diameters of approximately (c) 50 nm, (d) 100 nm, and (e) 150 nm for silica nanoparticles.

ASNPs were successfully synthesized by post-synthesis functionalization using APTES. ASNPs were dispersed in water and sonicated for size analysis using DLS. Figure 1a shows that mean modal diameters of 57 ± 3 nm (PDI 0.035), 108 ± 6 nm (PDI 0.095), and 153 ± 4 nm (PDI 0.029) were achieved with low polydispersity and high monodispersity through the control of the NH_4_OH concentration. Figure 1b shows that bare nanoparticles had a zeta potential of −37 mV due to the abundance of OH groups on the surface, which was due to the deprotonated silanol groups. ASNPs had a surface zeta potential of +27 mV due to the amine groups on the surface. TEM images in Figure 1c–e show low agglomeration of ASNPs post functionalization and confirmed the regularity of the nanoparticle’s shapes.

### Nanocomposite Double-Network Hydrogels

**Figure 2:**
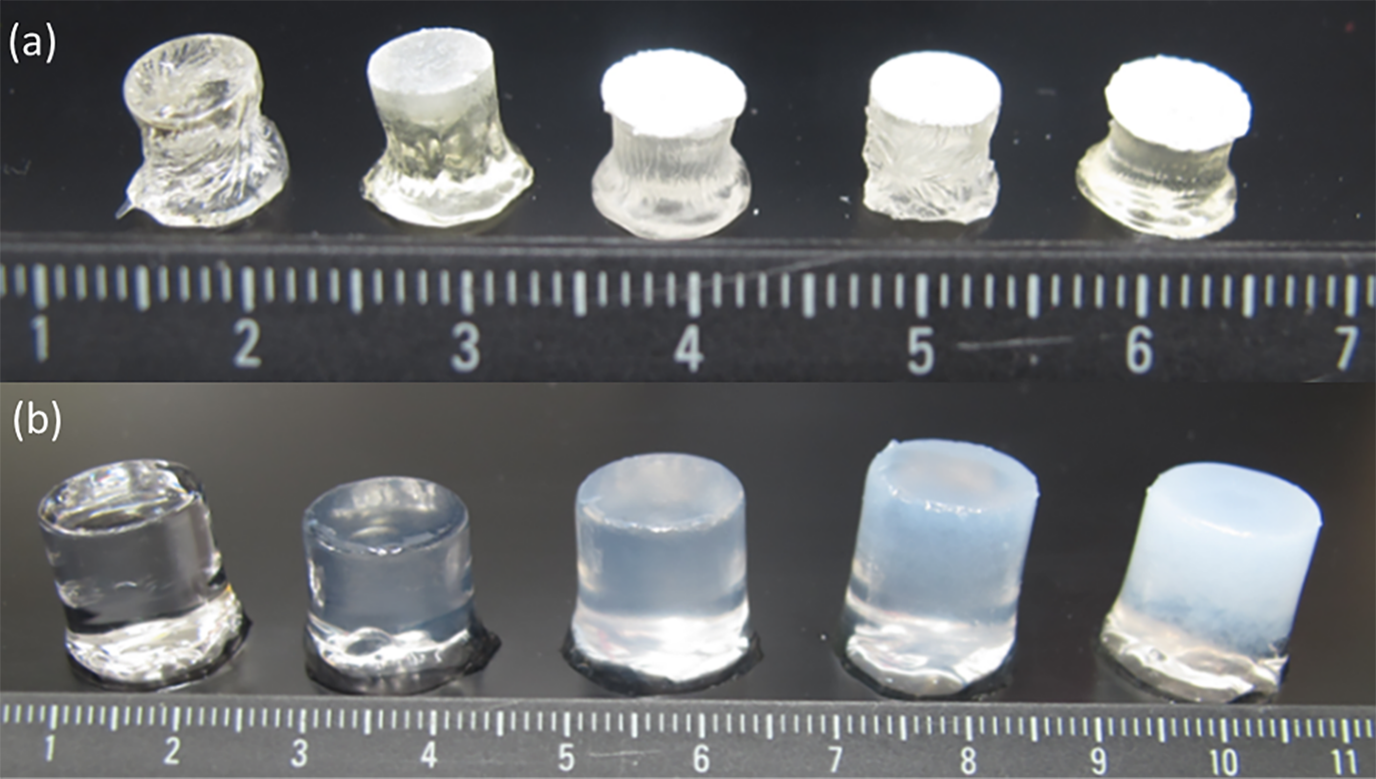
Images showing (a) freshly made PAAc/PAAm hydrogels before swelling and (b) PAAc/PAAm hydrogels after swelling for 150 h in DI-H_2_O. Hydrogels shown from left to right: control and 2.5, 10, 20, and 40 wt % loading with 150 nm amine silica nanoparticles (ASNPs).

The first-network gel was formed by cross-linking the carboxylic groups from PAAc and the amine group from the ASNPs via 1-ethyl-3-(3-dimethylaminopropyl)carbodiimide (EDC). PAAc was dissolved in MES buffer with EDC to form an unstable reactive ester (*O*-acylisourea). NHS was used to provide a more stable intermediate for reactions with primary amines from the ASNPs to form covalent amide bonds (Figure 7).^42,43^*O*-acylisourea, in theory, can react directly with primary amines without the need for an intermediate; however, the reaction rate is significantly low.^42,44^ The second network was successfully formed by swelling the ASNP-PAAc first-network gel in a solution of AAm monomers and photocuring the final hydrogels. The PAAc/PAAm formed showed a distinctive change in transparency based on the loading concentration of ASNPs with that at 40 wt % being the least transparent (Figure 2).

### Swelling

All hydrogels reached a plateau at approximately 150 h after being swollen in water. Previous research has shown that an increasing nanoparticle cross-linking density in the first network could result in hydrogels exhibiting a decrease in swelling properties.^45^ Water content values showed no significant changes with the introduction of ASNPs across all samples when compared to the control (Table S1). The water content ranged from 88–92%. Figure 2 shows the control and PAAc/PAAm hydrogels containing 150 nm ASNPs before and after being swollen. Hydrogels with 20 and 40 wt % ASNPs had a higher packing density in their upper half (Figure 2). This is attributed to ASNP sedimentation during gelation. This would have been the lower region when the gels were synthesized in the molds. However, gels with up to 10 wt % ASNPs are optically homogeneous after swelling, indicating that sedimentation or particle agglomeration may occur at higher concentrations.

**Figure 3:**
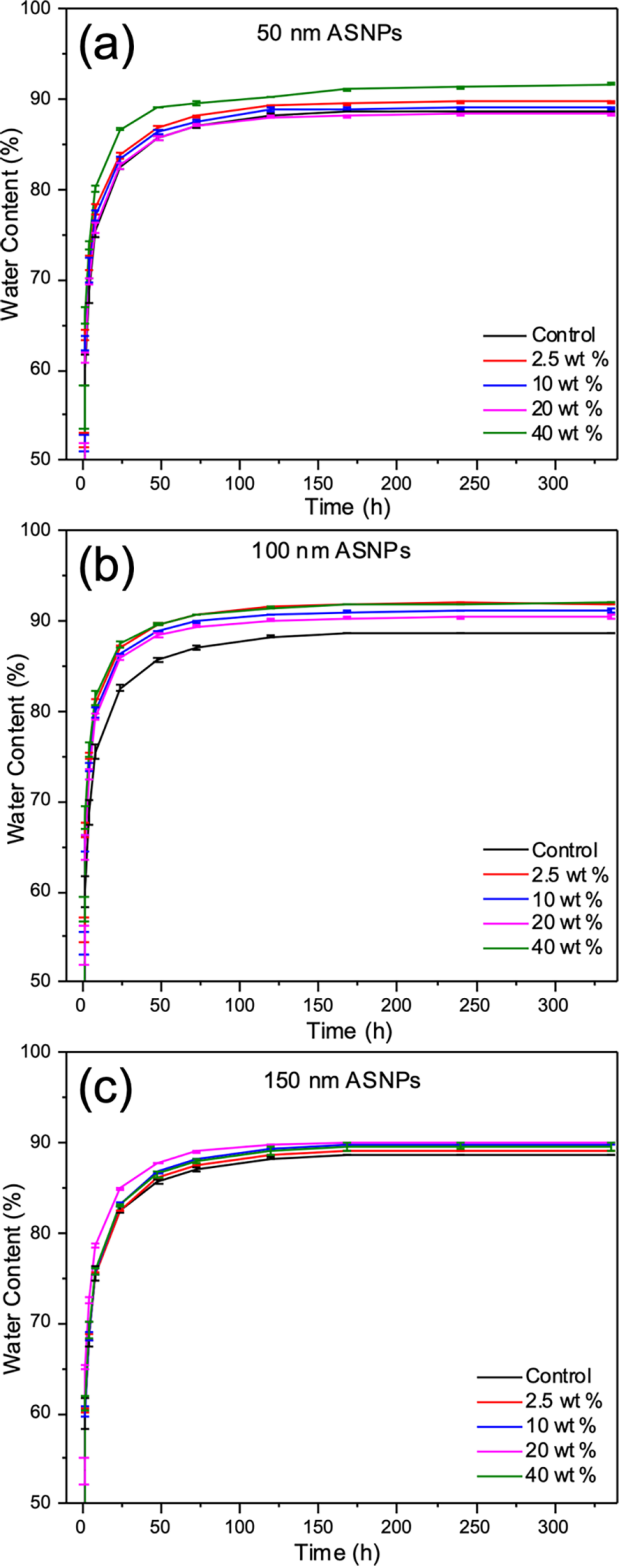
Swelling profiles for PAAc/PAAm hydrogels with (a) 50 nm, (b) 100 nm, and (c) 150 nm amine silica nanoparticles (ASNPs) at different loading concentrations compared to control.

Figure 3 shows that the hydrogels began to swell immediately when placed in water. This was mainly due to the hydrophilic nature of the two polymeric networks. The polymeric matrix becomes bound to water, which is known as “primary bound water”.^46^ Once the polymeric networks begin to swell, the hydrophobic groups are exposed. This results in hydrophobic bound water known as “secondary bound water”.^46^ The “total bound water”, which is the collective bound water of the primary and secondary states, is considered the initial swelling state. Due to the osmotic driving forces in the hydrogels, the polymeric networks continue to absorb water until an equilibrium state is reached. This equilibrium would be due to osmotic forces being canceled out by retraction forces of the swollen polymer networks. This additional water is considered “bulk water”^46^ and is the plateau of water absorption. Figure 3 shows the swelling data for hydrogels with 150, 100, and 50 nm ASNPs compared to the control. The highest value exhibited for water content was 91.96% ± 0.03 for hydrogels with 150 nm ASNPs at 2.5 wt % loading. The lowest water content value exhibited was 88.34% ± 0.04 for hydrogels with 50 nm ASNPs at 20 wt % loading. Swelling studies were conducted on multiple samples from the same synthesis batch, which accounts for the small standard error in the mean of each time point for the water content, as seen in Figure 3.

### FTIR

FTIR results showed that unreacted AAm monomers and non-cross-linked PAAc were present in the system and were washed out after swelling in water, along with ASNPs. Further analysis can be found in the Figure S1 and Table S2. The loss of ASNPs during swelling suggests that cross-linking may not have been complete for the first network or that excess ASNPs may be floating unbound and were therefore washed-out during swelling. It is not possible to quantify the mass of nanoparticles that remain in the PAAc/PAAm hydrogels through FTIR. Therefore, ASNP retention was calculated using thermogravimetric analysis (TGA) on swollen hydrogels.

### TGA

**Figure 4:**
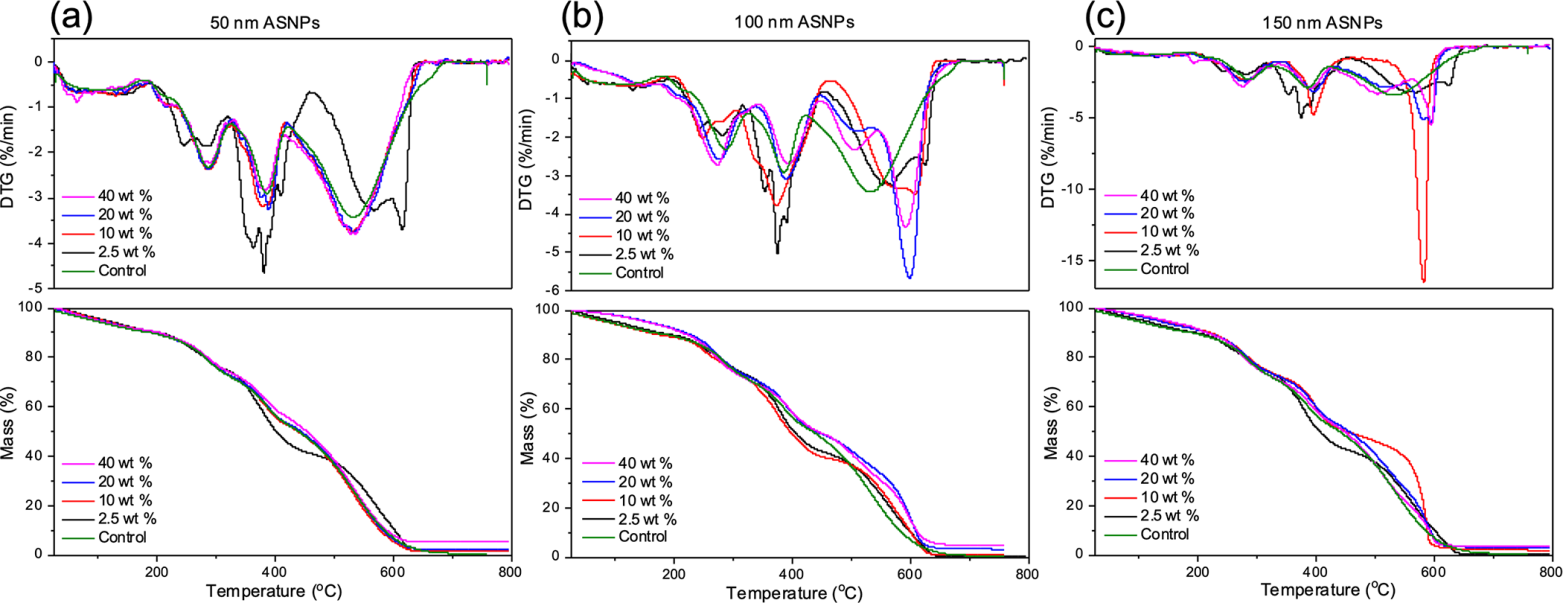
TGA showing nanoparticle retention for PAAc/PAAm hydrogels for (a) 50 nm, (b) 100 nm, and (c) 150 nm amine-functionalized silica nanoparticles (ASNPs) at different loading concentrations compared to the control.

Table 1 provides a summary of nanoparticle retention (NPR) values for the series of PAAc/PAAm hydrogels. The polymeric material in the samples was expected to be burnt off at 600 °C, and any remaining residues were considered to be silica nanoparticles. Figure 4a–c shows the mass loss profiles for PAAc/PAAm gels with 50, 100, and 150 nm ASNPs using TGA and DSC. Figure 4c shows the TGA profiles of PAAc/PAAm hydrogels with varying sizes and concentrations of ASNPs with their corresponding DTG profiles. As expected, control samples without ASNPs reached 100% mass loss at 630 °C with an NPR value of 0%. NPR was above 95% for all PAAc/PAAm hydrogels containing 150 nm ASNPs with the exception of gels with 40 wt % ASNPs, which only had 81% NPR. The lowered NPR value for hydrogels containing 40 wt % ASNPs could be due to ASNP leaking out during swelling. This is perhaps due to ASNPs that were not bonded to the polymer, ASNP loss during synthesis of the first network, or an inhomogeneous distribution of ASNPs due to a sinking effect of nanoparticles during gelation of the first network. If ASNPs are not cross-linked in the first network, they can potentially seep through the hydrogel pores while being soaked in the monomer solution of the second network. The 150 nm nanoparticles have smaller specific surface areas compared to 50 and 100 nm nanoparticles^47^ and therefore have less functional groups available for cross-linking per unit mass of ASNPs. The DTG curve for 10 wt % shows a shift to the right compared to the control, meaning that higher temperatures and more energy are required to break the bonds and burn off the material. This suggests that the 10 wt % ASNP loading had improved cross-linking relative to other concentrations. Figure 4a,b shows the TGA and DTG profiles for hydrogels containing 50 and 100 nm ASNPs, respectively. Hydrogels containing 100 nm ASNPs follow a similar trend to those of 150 nm with the exception of 10 wt % ASNPs, which only had 56% NPR. The increased loss can be due to the same reasons explained above. In general, hydrogels with a 100 nm ASNP loading had a lower NPR compared to that of 150 and 50 nm with the exception of gels containing 20 wt % 50 nm ASNPs (Figure 4 a). Hydrogels containing 50 nm ASNPs follow a similar trend to those of 100 nm and hydrogels containing 150 nm ASNPs. For all hydrogels containing 40 wt % ASNPs, the NPR was lower. Although it was expected that larger specific areas for 50 and 100 nm ASNPs would result in better nanoparticle retention, an effect of easier percolation could have caused higher NPR values. At a 40 wt % loading, the first network could be overloaded and oversaturated with nanoparticles that are not cross-linked and consequently washed out or lost during synthesis. Oversaturation of the first network at the highest ASNP concentration could have caused aggregates of ASNPs that resulted in the sinking effect that causes an inhomogeneous distribution (Figure 2). This was evident during synthesis as a longer sonication time was required for samples with a 40 wt % loading. These clusters of ASNPs aggregates are potentially detrimental to the mechanical properties of the hydrogels as they create voids in the sample that are uncross-linked. Ultimately, TGA and DTG show that there ASNPs were likely bonded with PAAc in the first network and that there were physical interactions within the two networks.

**Table 1:** Nanoparticle Retention for PAAc/PAAm Hydrogels for 50 nm Amine Silica Nanoparticles (ASNPs) at Different Loading Concentrations Compared to the Control

	2.5 wt %	10 wt %	20 wt %	40 wt %
Loading of 50 nm ASNPs				
theoretical %	0.42	1.67	3.33	6.67
residual %	0.39	1.48	2.00	5.10
particle retention %	94	89	60	76
Loading of 100 nm ASNPs				
theoretical %	0.42	1.67	3.33	6.67
residual %	0.34	0.94	3.09	4.66
particle retention %	82	56	93	70
Loading of 150 nm ASNPs				
theoretical %	0.42	1.67	3.33	6.67
residual %	0.40	1.60	3.33	5.40
particle retention %	95	96	100	81

### Mechanical Testing

**Figure 5:**
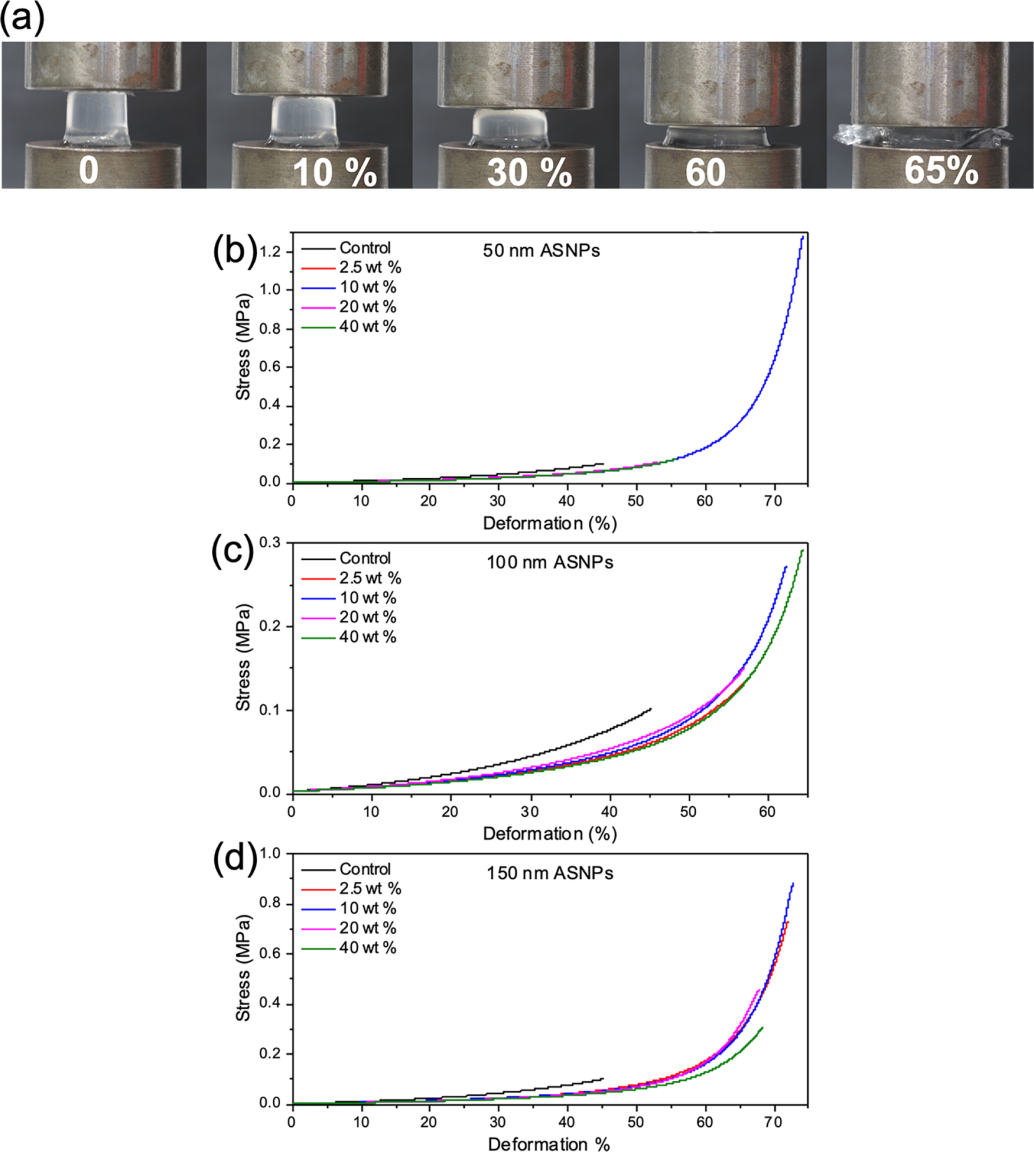
(a) Images of a 50 nm 10 wt % NC-DN hydrogel being compressed until failure. (b) Compression curves for PAAc/PAAm hydrogels with (b) 50 nm, (c) 100 nm, and (d) 150 nm amine silica nanoparticles (ASNPs) at different loading concentrations compared to the control.

Swollen PAAc/PAAm hydrogels were tested under uniaxial mechanical compression, as shown in Figure 5 and summarized in Table 2. The compressive stress at failure for controls was 0.10 ± 0.03 MPa at 45 ± 2% strain. Overall, ASNPs provided increased elasticity and allowed the material to be compressed up to 70% (Figure 5a), compared to control gels that failed at 40% deformation. Figure 5b shows representative compression curves for hydrogels containing 50, 100, and 150 nm ASNPs. Hydrogels with 2.5 wt % 50 nm ASNPs had little impact on the compressive failure strength, similar to 2.5 wt % 100 nm hydrogels. Again, this means that a combination of a low ASNP loading at 2.5 wt % and a smaller ASNP size of 50 nm does not have sufficient impact on hydrogel properties. Increasing the 50 nm ASNP loading to 10 wt % had a significant impact on failure at compression, that is, at 1.28 ± 0.26 MPa with 74 ± 13% strain, whereas hydrogels of 20 and 40 wt % 50 nm ASNPs had similar values again to those of the control.

**Table 2:**
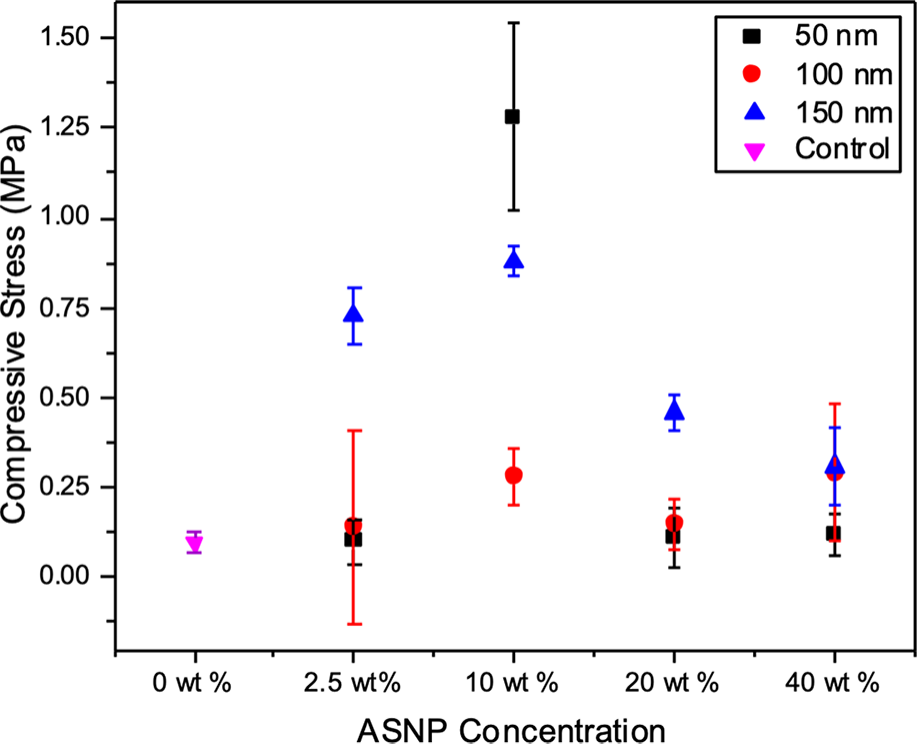
Compression and Strain for PAAc/PAAm Hydrogels with 50, 100, and 150 nm Amine Silica Nanoparticles (ASNPs) at Different Loading Concentrations Compared to the Control

	control	2.5 wt %	10 wt %	20 wt %	40 wt %
Loading of 50 nm ASNPs					
fracture compressive stress (MPa)	0.10 ± 0.03	0.10 ± 0.06	1.28 ± 0.26	0.11 ± 0.08	0.12 ± 0.06
fracture strain (%)	45 ± 2	52 ± 4	74 ± 13	53 ± 4	56 ± 3
Loading of 100 nm ASNPs					
fracture compressive stress (MPa)	0.10 ± 0.03	0.14 ± 0.02	0.27 ± 0.08	0.15 ± 0.07	0.29 ± 0.19
fracture strain (%)	45 ± 2	57 ± 10	62 ± 4	58 ± 2	64 ± 1
Loading of 150 nm ASNPs					
fracture compressive stress (MPa)	0.10 ± 0.03	0.73 ± 0.08	0.88 ± 0.04	0.46 ± 0.05	0.31 ± 0.11
fracture strain (%)	45 ± 2	72 ± 5	72 ± 4	67 ± 2	68 ± 5

For 150 nm ASNPS, the highest compressive stress at failure was at 0.88 ± 0.04 MPa and 72 ± 4% with 10 wt % ASNPs. The nanocomposite structure in the hydrogels had an impact on the mechanical properties of the hydrogel. This was likely due to the bonds between PAAc and ASNPs that held the polymer chains together while under stress, which were not present in control hydrogels. Figure 5b shows the compression curves for hydrogels containing 100 nm ASNPs exhibiting similar mechanical properties and responses to hydrogels containing 150 nm ASNPs.

A trend can be seen across all hydrogels where the 10 wt % loading in the first network provided the best results for increasing strain and failure. In comparison, the 2.5 wt % loading was too low to provide an impact on the mechanical properties, especially with sizes of 50 and 100 nm. Both 20 and 40 wt % loadings potentially resulted in unbound or non-cross-linked ASNPs, which may result in agglomeration of particles or non-cross-linked regions of the gel. ASNPs may agglomerate into clusters and cause an inhomogeneous distribution within the gel, resulting in a lack of covalent links between polymers and nanoparticles. This can result in reduced mechanical properties. This highlights that the desirable effects of ASNPs may be reached with lower concentrations in the range of 10 wt %.

Ultimately, the cross-linking chemistry of the first network is vital to the success of such tuning of the physical properties of PAAc/PAAm hydrogels. The combination effect of the nanoparticle concentration and size can impact the mechanical properties of the hydrogels. This is summarized and highlighted in Table 2. ASNP loading concentrations of over 2.5 wt %, particularly for 150 nm ASNPs, show enhanced mechanical strengths. It can also be concluded that the 10 wt % ASNP loading provides the best results in terms of mechanical properties for all three ASNP sizes tested.

### SEM

**Figure 6:**
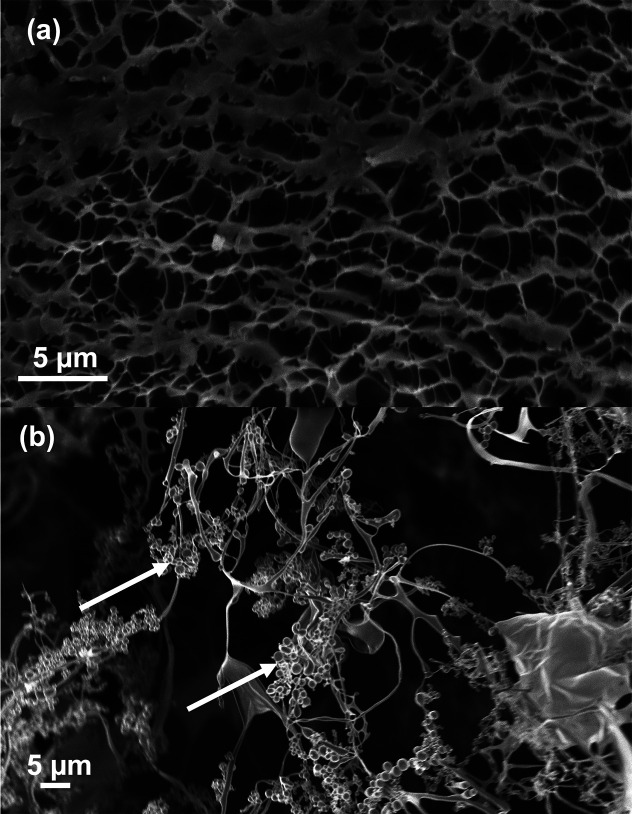
SEM images of (a) control PAAc/PAAm hydrogels without amine silica nanoparticles (ASNPs) and (b) PAAc/PAAm hydrogels with 150 nm amine silica nanoparticles (ASNPs) at a 10 wt % loading. ASNPs are highlighted with white arrows. Scale bars are set to 5 μm.

Hydrogel samples were frozen at a −80 °C overnight and freeze-dried for two days before SEM imaging. In Figure 6, cross-sections of the internal structure of control PAAc/PAAm hydrogels (Figure 6a) and PAAc/PAAm hydrogels with 10 wt % 150 nm ASNPs (Figure 6b) are shown. Figure 6a shows the absence of ASNPs in the control and the typical structure of a freeze-dried hydrogel. Figure 6b shows the interaction between polymeric structures and the ASNPs (highlighted with white arrows). In a, the pore-like structure is made of the entangled polymeric material that wraps around several ASNPs. This shows that ASNPs were able to integrate into the gel mesh. The nanocomposite structure integrated into the PAAc/PAAm hydrogels has shown to effectively improve the material’s mechanical strength. This is due to the entanglements between the PAAc network and good integration of ASNPS aided by the functionalization and efficient cross-linking. The SEM images provide insight into how ASNP–polymer networks could potentially fracture into clusters. These clusters have physical entanglements and chemical cross-links through the ASNPs with the PAAc/PAAm network and therefore provide the improved compressive toughness exhibited by the hydrogels. This has been demonstrated in previous works.^49^ Such topography and anchorage points could be useful for cell proliferation and growth in tissue-engineering applications.

## Conclusions

For the first time, novel nanocomposite double-network hydrogels were synthesized via carbodiimide coupling of amine-functionalized silica nanoparticles and polyacrylic acid as the first network. The second network was formed using polyacrylamide through photopolymerization. The final PAAc/PAAm hydrogels with ASNPs showed tailorable mechanical properties through the integration of nanoparticles with varying sizes and loading concentrations. This study proved the importance of the nanoparticle size and loading concentration and their impact on swelling and mechanical properties. SEM images revealed that ASNPs were integrated into the polymeric struts of the hydrogel mesh. The interaction of polymer chains with the ASNPs is believed to form an embedded micronetwork that improves the mechanical properties of the hydrogels with a maximum compressive stress of 1.28 MPa compared to 0.1 MPa of the control. The mechanical properties of these nanocomposite gels show an improvement to current nanocomposite IPN gels and are comparable to other nanocomposite DNHGs. Using different monomers, nanoparticles, and cross-linking chemistry, we were able to achieve similar properties to our previous work on ANC gels (1.78 MPa). The physical and chemical cross-links provide a key element for tailorability in these hydrogels and their application. These improvements in properties have broad applicability in biomedical sciences and tissue engineering such as in adhesives for wound healing, drug-delivery agents, implantable scaffolds, flexible sensors, and bio-wearables.

## Methods

### Double-Network Hydrogel Synthesis

**Figure 7:**
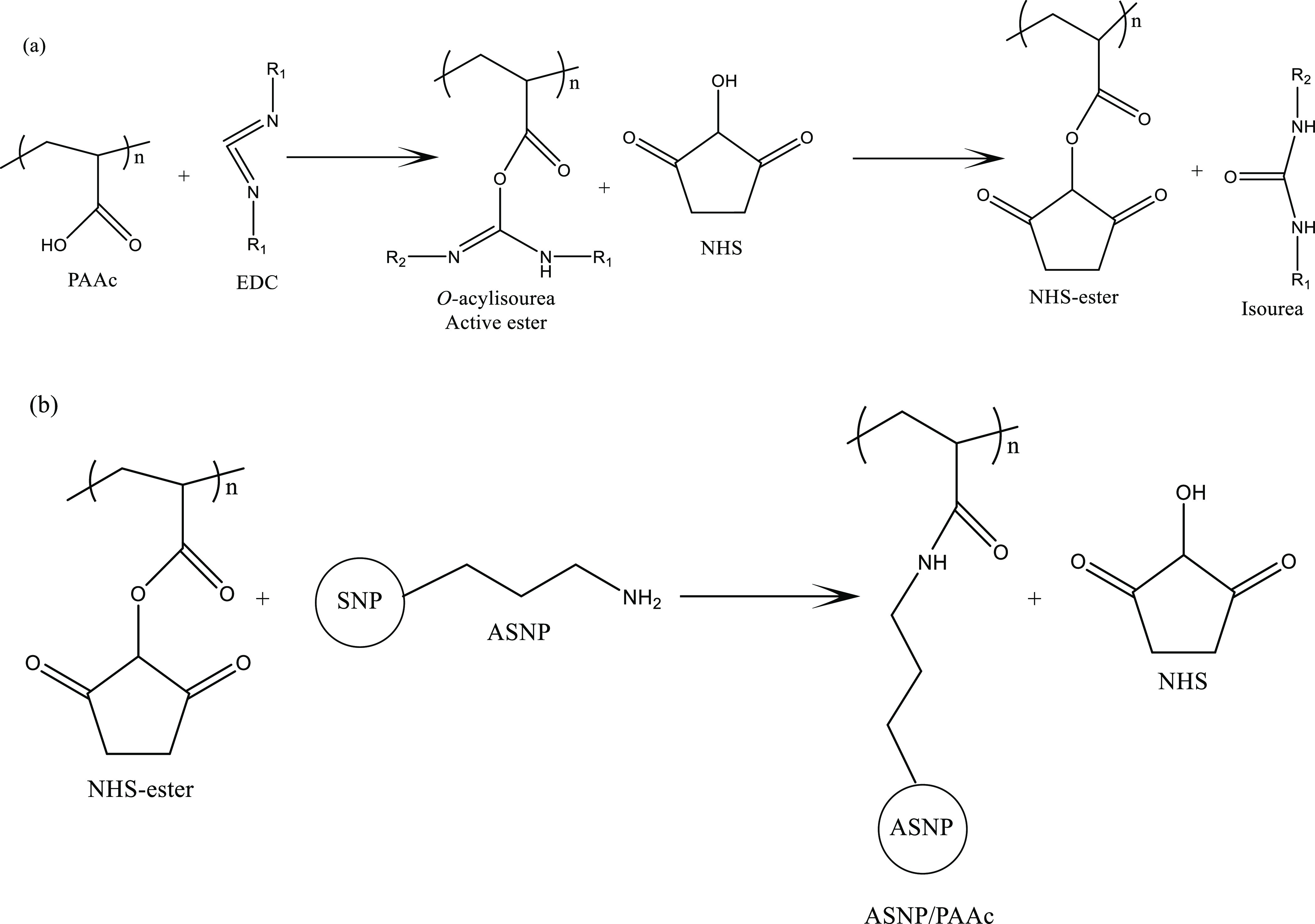
Schematic of the carbodiimide reaction between PAAc and EDC/NHS: (a) formation of a PAAc/NHS–ester and isourea byproduct and (b) cross-linking route between the intermediate PAAc/NHS–ester and amine-functionalized silica nanoparticles (ASNPs).

**Figure 8:**
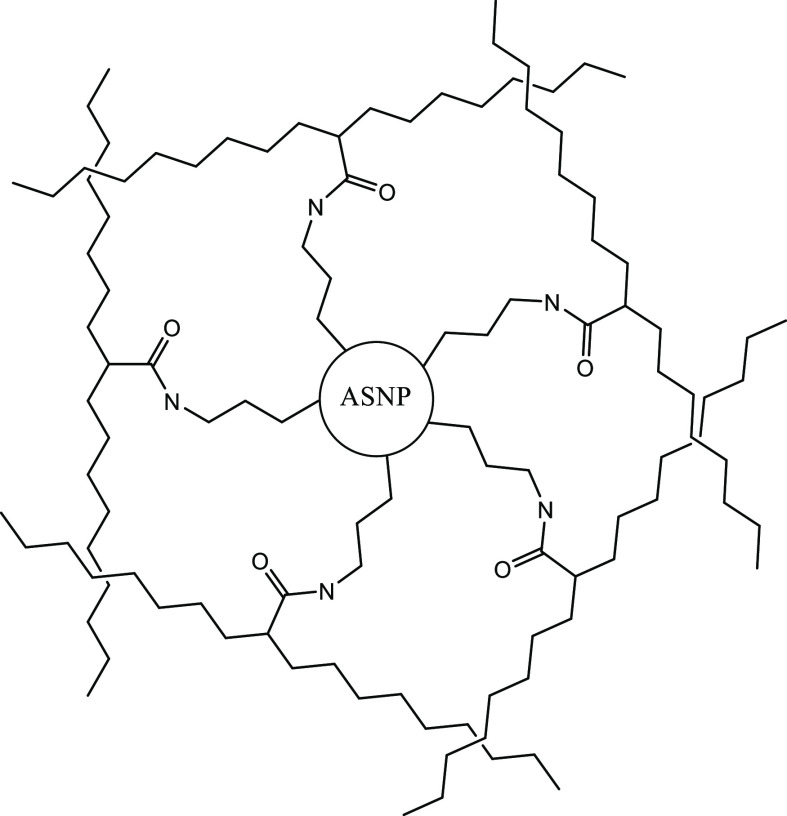
Schematic representation of a first-network PAAc system cross-linked with amine-functionalised silica nanoparticles.

Using carbodiimide chemistry, the tertiary −NH_2_ groups on the surface of the ASNPs (Figure 7) will form cross-links with the −COOH groups of polyacrylic acid (PAAc) to form a first-network gel (Figure 8). Successful cross-linking will allow a hydrogel to form by creating the first network, represented in Figure 8. The concentrations of carbodiimide components, namely, EDC and NHS, were chosen carefully to ensure that the intermediate NHS–ester formation takes place at the highest possible yield. This ensures that the reaction rate remains fast and efficient, preventing the formation of unwanted byproducts. Hence, the first-network gel was formed by cross-linking PAAc with ASNPs (Figure 7a,b). First, 2 mL of ready-made PAAc (25% solution in H_2_O; 50,000 M_w_), equivalent to 2 g, was dissolved in 4 mL of MES buffer solution at pH 5.5. The 2.5 wt % ASNPs with a 50 nm diameter relative to PAAc were dispersed in 1 mL of MES buffer and placed in a sonication bath for 30 min until fully sonicated. EDC was added in the powder form to the PAAc solution at a mass ratio of 1:2.7 to ASNP, and NHS was added at a mass ratio of 1:0.3 to EDC based on previous works and systemic experimental trials.^48−50^Table 3 shows the varying ASNP concentrations with equivalent carbodiimide reagent concentrations. The weights used for ASNP concentrations are the same across their respective concentrations for the various sizes, that is, 2.5 wt % 50 nm ASNPs will have the same weight as 2.5 wt % 100 and 150 nm ASNPs. The sonicated ASNPs were added when the solution was fully dissolved and left to stir until the solution became viscous. The solution was then poured into a polystyrene 48 cell-culture well plate, which acted as a mold. This was left for 6 h to allow for further cross-linking to occur between the ASNPs and PAAc polymer chains (Figure 8). Once the hydrogels were formed, they were carefully removed from the well plate and prepared to be soaked in an AAm monomer solution.

**Table 3:** Varying ASNP Concentrations with Equivalent Carbodiimide Reagent Concentrations^a^

ASNP (wt %)	ASNP (mg)	EDC (g)	NHS (g)
0	0	0	0
2.5	12.5	0.03	0.01
10	50	0.13	0.04
20	100	0.27	0.08
40	200	0.53	0.16

a The weights shown in the table apply to 50, 100, and 150 nm ASNPs.

**Figure 9:**
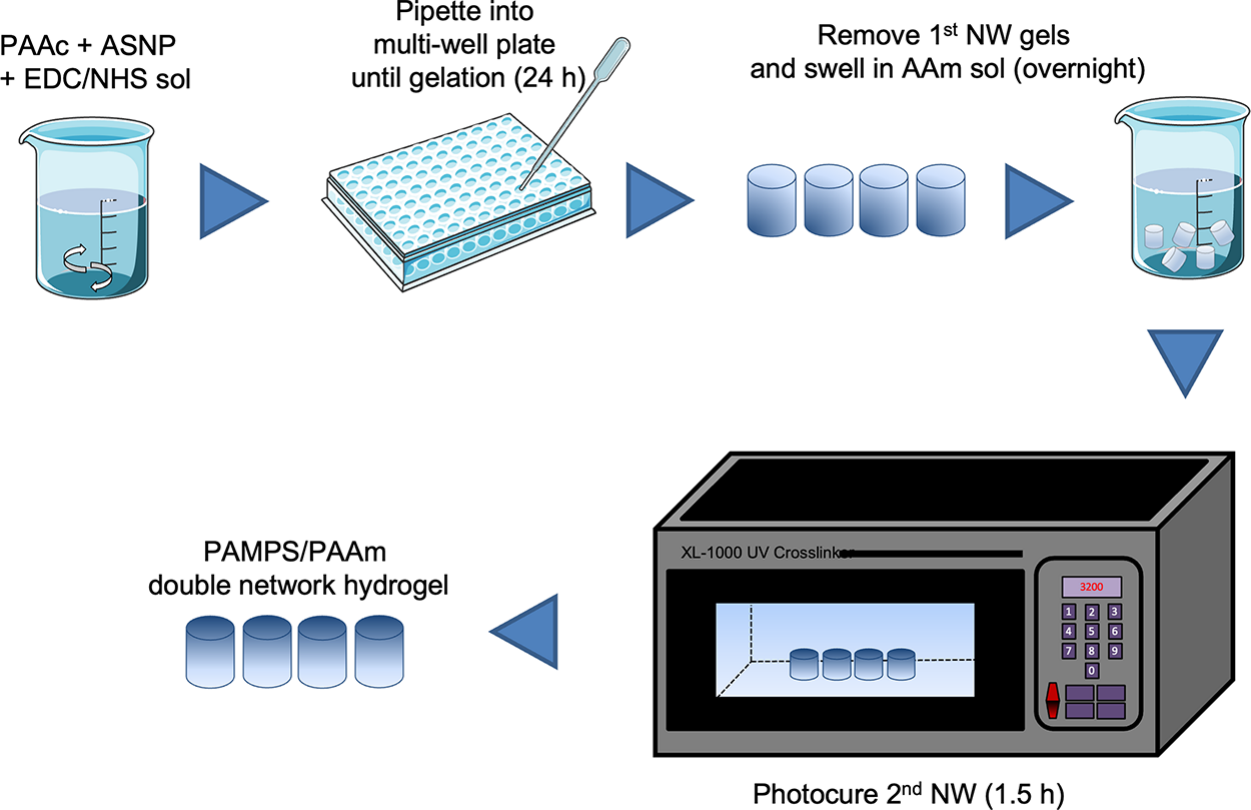
Schematic representation showing the synthesis route for double-network hydrogels (DNHGs) containing amine-functionalized silica nanoparticles (ASNPs).

Next, the second network was formed using AAm. An amount of 2.5 g of AAm, 1 wt % (25 mg) Irgacure 2959, and 1 wt % (25 mg) BIS were dissolved in 5 mL of deionized H_2_O. The hydrogels were placed in the AAm monomer solution and left until they were swollen. Once the hydrogels were swollen, they were placed in the UV cross-linker for photopolymerization of the second network to form the double-network hydrogel. The UV cross-linker was set to 5400 s at maximum UV intensity (average 2900 μW/cm^2^) of 365 nm of light. This 5400 s was found to be sufficient time for gelation. The double-network hydrogel is then removed and dried at 60 °C before being immersed in water for 14 days. The process was repeated for 2.5, 10, 20, and 40 wt % for 50, 100, and 150 nm ASNPs. A control DNHG was formed following the same procedure without ASNPs, EDC, and NHS. The synthesis route is depicted in Figure 9.

Hydrogels with varying ASNP sizes and compositions were dried at 60 °C directly post synthesis for five days to reach a fully dry state. Subsequently, the dried hydrogels were ground into a powder and used for characterizing the chemical content post synthesis, referred to as “fresh” samples. Samples from each batch were also placed in water directly post synthesis to be swollen for up to 14 days. Compression studies were conducted on the fully swollen hydrogels once the swelling study was completed. Swollen hydrogels are referred to as “swollen” samples and were also used for TGA and FTIR analysis.

## Supporting Information Available

Table of water content for PAAc/PAAm hydrogels vs control after swelling, FTIR spectra of fresh PAAc/PAAm hydrogels with 150 nm ASNPs at different loading concentrations vs control and fresh vs swollen samples of PAAc/PAAm hydrogels with 150 nm amine silica nanoparticles at 2.5 wt % loading, summary of FTIR bands for PAAc/PAAm hydrogels, list of materials, methods for synthesis of amine-functionalized silica nanoparticles, and characterization techniques including swelling, thermogravimetric analysis, mechanical testing, FTIR, and SEM (PDF)
