# Supplementary material for: Double-Network Hydrogels Reinforced with Covalently Bonded Silica Nanoparticles via 1-Ethyl-3-(3-dimethylaminopropyl)carbodiimide Chemistry
Source: ACS Omega. 2022 Nov 18;7(48):43904–14. doi: 10.1021/acsomega.2c05169 (PMC9730475; doi:10.1021/acsomega.2c05169)
Supplement: Supplementary file 1 — ao2c05169_si_001.pdf [file ao2c05169_si_001.pdf]

Double network hydrogels reinforced with covalently bonded silica nanoparticles via EDC chemistry

Ali A. Mohammed [1, 2], Nicholas Groth Merrild [2], Siwei Li [2,3], Alessandra Pinna [2, 4], Julian R. Jones[2, *]

[1] Dyson School of Design Engineering, Imperial College London, SW7 9EG

[2] Department of Materials, Imperial College London, SW7 2AZ, London, UK

[3] Visiting Specialist Services Academy Ltd, Office 6.072 6th Floor, First Central 200, 2 Lakeside Drive London NW10 7FQ

[4] The Francis Crick Institute, London NW11AT, UK

Supporting information

Table S1. Water content at 336 h of PAAc/PAAm hydrogels with 50 nm, 100 nm and 150 nm amine silica nanoparticles (ASNPs) with varying loading concentrations, compared to a control.

Loading of 50 nm ASNPs	Control	2.5 wt.%	10 wt.%	20 wt.%	40 wt.%
Water Content (%)	88.67 ± 0.03	89.73 ± 0.04	89.05 ± 0.06	88.34 ± 0.04	91.72 ± 0.21

Loading of 100 nm ASNPs	Control	2.5 wt.%	10 wt.%	20 wt.%	40 wt.%
Water Content (%)	88.67 % ± 0.03	89.17 % ± 0.03	89.91 % ± 0.03	90.06 % ± 0.07	89.64 % ± 0.46

Loading of 150 nm ASNPs	Control	2.5 wt.%	10 wt.%	20 wt.%	40 wt.%

Water Content (%)	88.67 % ± 0.03	91.96 % ± 0.02	91.17 % ± 0.14	90.48 % ± 0.14	92.04 % ± 0.04

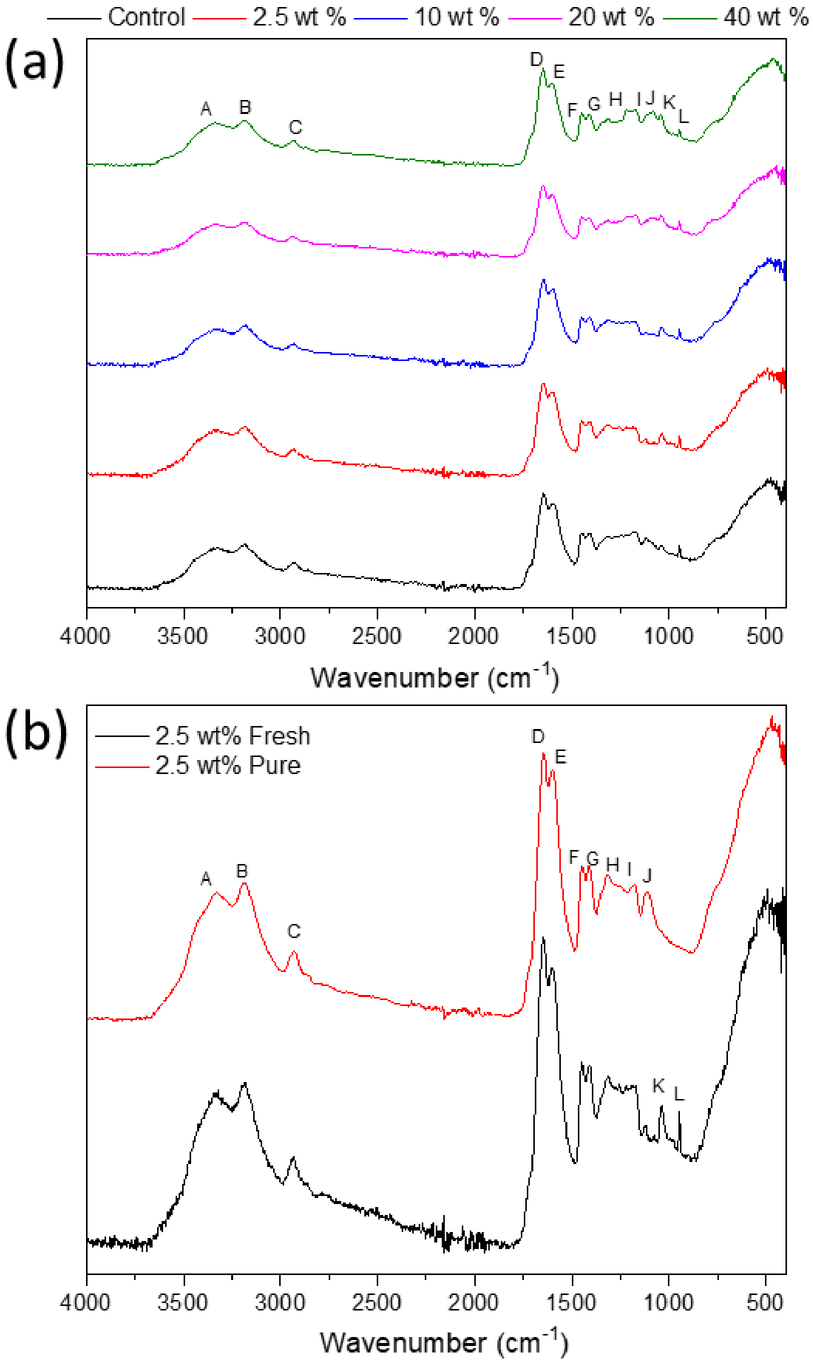

Figure S1. FTIR spectra of (a) Fresh PAAc/PAAm hydrogels with 150 nm silica nanoparticles (ASNPs) at different loading concentrations compared to control. (b) Fresh and

Swollen samples of PAAc/PAAm hydrogels with 150 nm amine silica nanoparticles (ASNPs) at 2.5 wt. % loading.

Table S2. Summary of FTIR bands for PAAc/PAAm hydrogels.

	Bond	Type	Wavenumber (cm^{-1})
A	N-H	Stretching	3324
B	O-H	Water remaining in sample	3082
C	C-H	Alkyl stretching	2950
D	C=O	Stretching, coupled with N-H bending	1650
E	N-H	Primary amine	1620
F	C-N	Stretching primary amide	1420
G	C-H	Deformation	1350
H	N-H	Bending	1238
H	C-O	Carboxylic acid stretch	1240
I	Si-O-Si	Silica nanoparticles	1160
J	NH_2	In-plane rocking	1120
K	C-OH	Side group vibrations	1043
L	O-H	Carboxylic acid stretch	943

FTIR was carried out on Fresh and Swollen samples with bands summarised in Supplementary Information: Table 2. Supplementary Information: Figure 1 (a) shows the stacked FTIR spectra for Fresh samples of hydrogels containing 150 nm ASNPs. Carboxylic groups (-COOH) bands are 1240 cm^{-1}, 1043 cm^{-1}, and 943 cm^{-1} [1-3]. These bands show the presence and integration of PAAc into the double network hydrogel. Bands of 1650 cm^{-1}, 1420 cm^{-1}, 3324 cm^{-1}, and 3082 cm^{-1} were generated by C=O stretching coupled with N-H bending, C-N stretching coupled with N-H bending, and medium to weak recordings of N-H stretching, respectively[4, 5]. N-H bending is also found at 1238 cm^{-1} [5]. These bands are produced due to the presence of PAAm in the hydrogel. Polymer backbones are found in the band of 2950 cm^{-1} which represents

vibrations of C-H alkyl stretching[6, 7]. C=C vibrations at 1635 cm^{-1} – 1676 cm^{-1} and 822 cm^{-1} give evidence of unreacted AAm monomers[8-10]. The intensity of these vibrations should decrease with higher polymer conversion, although it is difficult to conclude due to overlapping bands with C=O stretching coupled with N-H bending. Si-O-Si are found at 1160 cm^{-1} indicating presence of ASNPs[11]. Figure S1 (b) shows the difference between Fresh and Swollen 2.5 wt. % of hydrogels containing 150 nm ASNPs. A clear decrease for O-H carboxylic acid stretch at 943 cm^{-1} was seen. This indicates that unbound PAAc chains were removed during swelling. A decrease in intensity of band 1160 cm^{-1} indicates that ASNPs could potentially have been lost during swelling. PAAm chains may have also been washed out of the hydrogel, due to the decreased intensity of C-H in 2950 cm^{-1}. The decrease in absorbance at 1050 cm^{-1} due to leaked PAAc chains allows the NH_2 band at 1120 cm^{-1} to become more prominent in the Swollen spectra.

Materials and Methods

Polyacrylic Acid (PAAc; 50,000 MW) was purchased from Polysciences Inc. USA. N-(3-dimethylaminopropyl)-N'-ethylcarbodiimide hydrochloride (EDC) was purchased from Alfa Aesar UK. N-hydroxysuccinimide (NHS), ethanol (EtOH; 200 proof anhydrous; ≥ 99.5 %); acrylamide (AAm; ≥ 99 %), photoinitiator 2-hydroxy-4′-(2-hydroxyethoxy)-2-methylpropiophenone (synonym Irgacure 2959; 98 %), ammonium hydroxide (28-30 % NH_3 basis), tetraethyl orthosilcate (TEOS; 98 %), (3-aminopropyl) triethoxysilane (APTES; ≥ 98 %) and N, N'-methylenebisacrylamide (BIS; 99 %) were purchased from Sigma-Aldrich (UK). No additional processing or purifications were performed.

Amine functionalized silica nanoparticle (ASNPs) synthesis

Silica nanoparticles (SNPs) were synthesized based on the Stöber process to produce monodispersed SNPs[12-15]. SNPs were synthesized by using fixed molar concentrations of TEOS (0.28 M) and water (6 M) and adjusting ammonium hydroxide (NH_4OH) to increase or decrease the diameter of SNPs. SNPs with a diameter of 150 nm were synthesized by measuring out 82.28 ml EtOH and 10.27 ml deionised H_2O into a beaker. 0.3 M NH_4OH (1.19 ml) was added to the solution followed directly by 6.25 ml TEOS as identified by Greasley *et al*[15]. The solution was left to stir at 500 rpm for 24 h. The final milky white solution was centrifuged using an Eppendorf 5430 and washed with EtOH three times at 7,830 rpm to remove any unreacted TEOS. The final precipitate was left to dry at 60 ^oC overnight. SNPs with 50 nm diameter were synthesized using NH_4OH 0.2 M, and SNPs with 100 nm diameter was synthesized using NH_4OH 0.28 M. Figure S2 (a) shows the reaction steps for SNP synthesis.

Next, the nanoparticles were functionalised with primary amine groups by dispersing 500 mg of dried SNPs in 10 ml EtOH and sonicated using the CamSonix 1800T until a homogenous dispersion was achieved. The solution was topped up with 90 ml EtOH whilst stirring. 500 µl anhydrous NH_4OH and 3 ml APTES were added to the solution and left to stir for a further 24 h. Once the reaction was complete, the solution was centrifuged and washed three times with EtOH to remove any unreacted APTES. The final precipitate was dried at 60 ^oC and stored under dry conditions. Figure S2 (b) demonstrates the surface functionalization of SNPs using APTES.

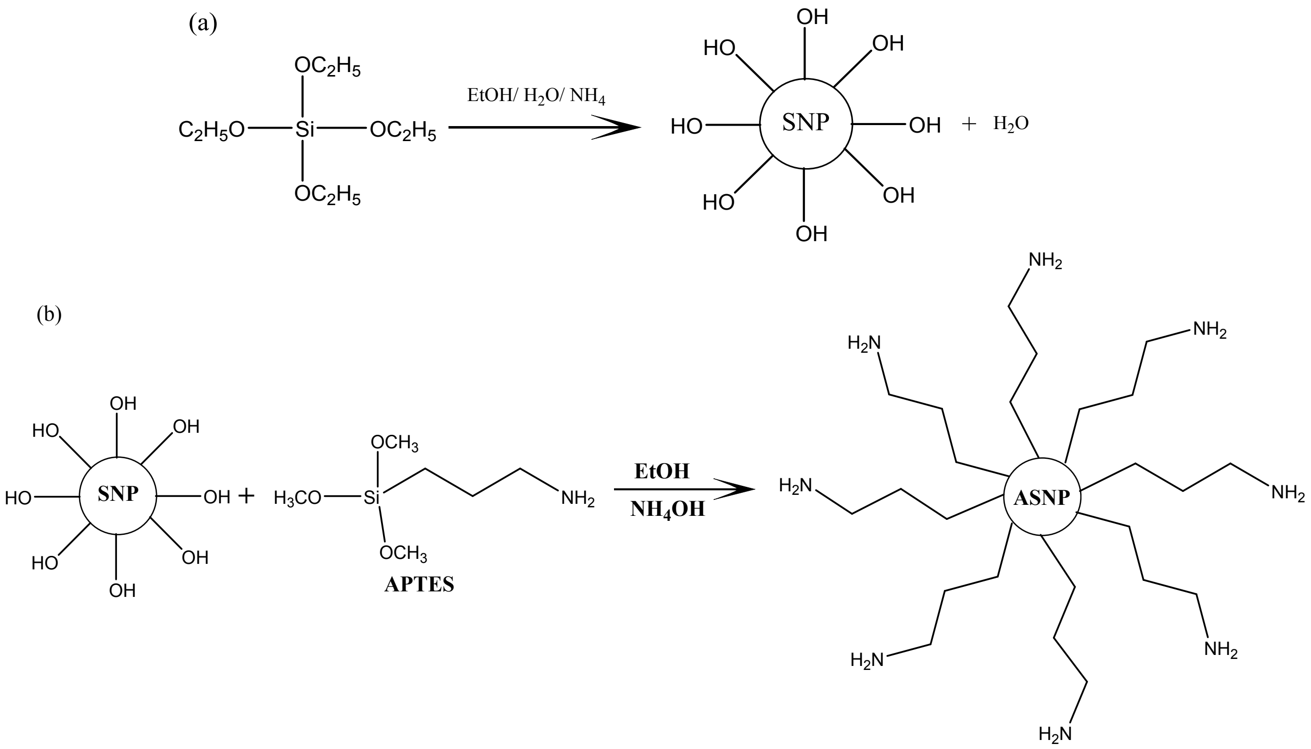

Figure S2. Schematic of (a) Synthesis of silica nanoparticles (SNPs) using the Stöber process; (b) followed by surface functionalization of SNPs by (3-aminopropyl) triethoxysilane (APTES) to produce amine functionalised silica nanoparticles (ASNPs).

Characterization

Hydrogels were placed in DI-H_2O post-synthesis and weighed at 0 h, 1 h, 2 h, 4 h, 8 h, 24 h, 48 h, 72 h, 120 h, 168 h, 240 h and 336 h. Water content was calculated using Equation (1):

$$\text{Water content (\%)} = \frac{M_{s(t)} - M_d}{M_{s(t)}} \qquad \text{Equation (1)}$$

Where $M_{s(t)}$ is the swollen mass of the sample at time (t), and M_d is the initial dry mass of the sample.

SNPs were diluted in ethanol, sonicated and then carefully dropped onto 300 mesh copper TEM grids coated with holey carbon film. A JEOL FX2000 was used for imaging with tungsten filament as the electron source at an operating voltage of 200 kV and a 10 μm objective aperture. Mean diameter was calculated by measuring 100 separate SNPs.

DLS sample volumes of 100 μl were diluted in 1 – 1.5 ml of DI-H_2O and sonicated using the CamSonix 1800T until the nanoparticles were suspended. Glass quartz cuvettes were used for analysis in the Nano ZS ZEN3600 Zetasizer, and each sample was run 3 times with 12 scans.

TGA was carried out for all Swollen samples to calculate nanoparticle retention (NPR) using Equation (2). Swollen samples were dried for 5 days at 60 ^{o}C and ground to a powder. Polymeric material in the samples was expected to be burnt off by 600 ^{o}C and any remaining residues are considered to be SNPs.

Equation (2)

$$\mathrm{NPR}\ (\%) = \frac{\text{residual mass } (\%)}{\text{theoretical mass } (\%)} \times 100\% = \frac{\text{residual mass } (\%)}{\mathrm{M_p} / (\mathrm{M_p} + \mathrm{M_1} + \mathrm{M_2})} \times 100\%$$

Swollen samples were used for mechanical analysis in compression using a Zwick Roell z2.5 machine fitted with a load cell of 10 kN and a strain rate of 1.5 mm min^{-1}. Each ASNP concentration per ASNP size was tested with 5 samples until failure. An average compressive stress and strain value with their respective standard error were calculated using these repeats.

FTIR samples were dried at 60 ^{o}C and ground to a fine powder for prior to analysis. A Nicolet iS10 Thermo Scientific FTIR was used. Samples were scanned 32 times per run with a resolution of 6 in a wavenumber region of 4000 – 400 cm^{-1}.

Samples for SEM were frozen overnight at -80 °C and then freeze dried for 24 h. Samples were placed on SEM sample holders and held by carbon tape. Samples were then sputter coated for 2 minutes at 20 mA to form a 10 nm layer of chromium. The LEO Gemini 1525 FEG-SEM was used to image the surface morphology of freeze-dried hydrogels using 3 – 5 kV and a working distance of 5 – 10 mm.

References

1. Li, A.; Wang, A.; Chen, J., Studies on poly(acrylic acid)/attapulgite superabsorbent composite. I. Synthesis and characterization. *J. Appl. Polym. Sci.* **2004,** *92* (3), 1596-1603.

2. Smitha, B.; Sridhar, S.; Khan, A. A., Polyelectrolyte Complexes of Chitosan and Poly(acrylic acid) As Proton Exchange Membranes for Fuel Cells. *Macromolecules* **2004,** *37* (6), 2233-2239.

3. Peniche, C.; Argüelles-Monal, W.; Davidenko, N.; Sastre, R.; Gallardo, A.; San Román, J., Self-curing membranes of chitosan/PAA IPNs obtained by radical polymerization: preparation, characterization and interpolymer complexation. *Biomaterials* **1999,** *20* (20), 1869-1878.

4. Wang, X. H.; Li, D. P.; Wang, W. J.; Feng, Q. L.; Cui, F. Z.; Xu, Y. X.; Song, X. H.; van der Werf, M., Crosslinked collagen/chitosan matrix for artificial livers. *Biomaterials* **2003,** *24* (19), 3213-3220.

5. Ma, D. H.-K.; Lai, J.-Y.; Cheng, H.-Y.; Tsai, C.-C.; Yeh, L.-K., Carbodiimide cross-linked amniotic membranes for cultivation of limbal epithelial cells. *Biomaterials* **2010,** *31* (25), 6647-6658.

6. Iwasita, T.; Pastor, E., A DEMS AND FTIR SPECTROSCOPIC INVESTIGATION OF ADSORBED ETHANOL ON POLYCRYSTALLINE PLATINUM. *Electrochim. Acta* **1994,** *39* (4), 531-537.

7. Maeda, Y.; Higuchi, T.; Ikeda, I., Change in Hydration State during the Coil−Globule Transition of Aqueous Solutions of Poly(N-isopropylacrylamide) as Evidenced by FTIR Spectroscopy. *Langmuir* **2000,** *16* (19), 7503-7509.

8. Ngadaonye, J. I.; Cloonan, M. O.; Geever, L. M.; Higginbotham, C. L., Synthesis and characterisation of thermo-sensitive terpolymer hydrogels for drug delivery applications. *J. Polym. Res.* **2011,** *18* (6), 2307-2324.

9. Ferreira, P.; Coelho, J. F. J.; Gil, M. H., Development of a new photocrosslinkable biodegradable bioadhesive. *Int. J. Pharm.* **2008,** *352* (1), 172-181.

10. Wang, Q.; Hou, R.; Cheng, Y.; Fu, J., Super-tough double-network hydrogels reinforced by covalently compositing with silica-nanoparticles. *Soft Matter* **2012,** *8* (22), 6048-6056.

11. Beganskienė, A.; Sirutkaitis, V.; Kurtinaitienė, M.; Juškėnas, R.; Kareiva, A., FTIR, TEM and NMR investigations of Stöber silica nanoparticles. *Mater Sci (Medžiagotyra)* **2004,** *10*, 287-290.

12. Stöber, W.; Fink, A.; Bohn, E., Controlled growth of monodisperse silica spheres in the micron size range. *J. Colloid Interface Sci.* **1968,** *26* (1), 62-69.

13. Bagwe, R. P.; Hilliard, L. R.; Tan, W., Surface Modification of Silica Nanoparticles to Reduce Aggregation and Nonspecific Binding. *Langmuir* **2006,** *22* (9), 4357-4362.

14. Qhobosheane, M.; Santra, S.; Zhang, P.; Tan, W., Biochemically functionalized silica nanoparticles. *Analyst* **2001,** *126* (8), 1274-1278.

15. Greasley, S. L.; Page, S. J.; Sirovica, S.; Chen, S.; Martin, R. A.; Riveiro, A.; Hanna, J. V.; Porter, A. E.; Jones, J. R., Controlling particle size in the Stöber process and incorporation of calcium. *J. Colloid Interface Sci.* **2016,** *469*, 213-223.
